# Characterization of novel SSR markers in diverse sainfoin (*Onobrychis viciifolia*) germplasm

**DOI:** 10.1186/s12863-016-0431-0

**Published:** 2016-08-30

**Authors:** Katharina Kempf, Marina Mora-Ortiz, Lydia M. J. Smith, Roland Kölliker, Leif Skøt

**Affiliations:** 1 Agroscope ISS, Reckenholzstrasse 191, CH-8046 Zürich, Switzerland; 2 National Institute of Agricutural Botany, Huntingdon Road, Cambridge, CB3 OLE UK; 3 IBERS, Aberystwyth University, Gogerddan, Aberystwyth, Ceredigion SY23 3EB UK

**Keywords:** *Onobrychis viciifolia*, Sainfoin, Microsatellite, SSR, Genetic diversity, Molecular markers, Fingerprinting

## Abstract

**Background:**

Sainfoin is a perennial forage legume with beneficial properties for animal husbandry due to the presence of secondary metabolites. However, worldwide cultivation of sainfoin is marginal due to the lack of varieties with good agronomic performance, adapted to a broad range of environmental conditions. Little is known about the genetics of sainfoin and only few genetic markers are available to assist breeding and genetic investigations. The objective of this study was to develop a set of SSR markers useful for genetic studies in sainfoin and their characterization in diverse germplasm.

**Results:**

A set of 400 SSR primer combinations were tested for amplification and their ability to detect polymorphisms in a set of 32 sainfoin individuals, representing distinct varieties or landraces. Alleles were scored for presence or absence and polymorphism information content of each SSR locus was calculated with an adapted formula taking into account the tetraploid character of sainfoin. Relationships among individuals were visualized using cluster and principle components analysis. Of the 400 primer combinations tested, 101 reliably detected polymorphisms among the 32 sainfoin individuals. Among the 1154 alleles amplified 250 private alleles were observed. The number of alleles per locus ranged from 2 to 24 with an average of 11.4 alleles. The average polymorphism information content reached values of 0.14 to 0.36. The clustering of the 32 individuals suggested a separation into two groups depending on the origin of the accessions.

**Conclusions:**

The SSR markers characterized and tested in this study provide a valuable tool to detect polymorphisms in sainfoin for future genetic studies and breeding programs. As a proof of concept, we showed that these markers can be used to separate sainfoin individuals based on their origin.

**Electronic supplementary material:**

The online version of this article (doi:10.1186/s12863-016-0431-0) contains supplementary material, which is available to authorized users.

## Background

*Onobrychis viciifolia* Scop., commonly known as sainfoin, belongs to the tribe *Hedysareae* and the family *Fabaceae*. It is a tetraploid (2n = 4x = 28) perennial forage legume, rich in proteins and secondary plant metabolites. Its center of origin is attributed to the Middle East and Central Asia. It was introduced into Europe in the fifteenth century and was rapidly adopted by farmers due to its high fodder value, especially for working horses [1]. Nowadays, sainfoin is cultivated only in small areas for fodder production and on ecological compensation areas. Its cultivation steadily declined since the 1950’s, due to the expanding availability of inorganic fertilizers and the preference for higher yielding legume crops such as alfalfa (*Medicago sativa*) or red clover (*Trifolium pratense*) [2–4]. In the last few years, however, sainfoin has gained renewed interest due to its animal health promoting properties associated with the presence of condensed tannins (CT) and other complex phytochemicals in the foliage. Benefits include anthelminthic properties and prevention of potentially lethal bloat associated with most other forage legumes [5–9]. In addition, sainfoin shows a range of beneficial agronomic features. In common with most other legumes sainfoin fixes atmospheric nitrogen in its root nodules, thus reducing the need for industrial N fertilizer input. [1, 10]. Furthermore, soil fertility is improved by increased humus development through its deep rooting capability and low input requirements once established [11]. Used as a component of permanent grassland, sainfoin is a valuable alternative for areas suffering from intensification, as it increases soil fertility and has become a popular addition to non-cropped environmental planting; sainfoin provides good resources for native insects and high quality fodder for livestock [11].

Despite its advantages, a wide distribution of sainfoin is hampered by the often poor agronomic performance and the lack of sainfoin varieties adapted to different environmental conditions. The main weaknesses of sainfoin lie in its low tolerance to waterlogging and frost as well as in its poor competitive ability in the early stages of development. Therefore, targeted breeding activities are needed to select for sainfoin individuals better adapted to a broad range of environmental conditions. Breeding activities have also been impaired by the lack of knowledge of the genetic diversity of the species and its mode of inheritance. Further investigation and development of tools for marker assisted breeding has been hampered by the limited availability of species-specific molecular markers. So far, most studies have focused on the use of cross-amplifiable EST-SSRs, mainly from *Medicago* and *Glycine* species; ITS markers based on nuclear internal transcribed spacer regions and dominant SRAP markers [12–15]. The use of co-dominant SSR markers developed in other species yielded only a low number of alleles per locus in sainfoin (from 5 to 7 in bulks of 10 individual plants [12]. The development of highly informative, specific markers for sainfoin is indispensable to create a genetic knowledge base and assist breeding by marker assisted selection (MAS) [16].

SSRs or Microsatellites [17] are composed of tandemly repeated sections of DNA [18]. SSR markers show co-dominance of alleles and are randomly distributed along the genome, particularly in low-copy regions [19, 20]. Considering the complex tetraploid sainfoin genome and the lack of knowledge about its genetics, SSRs are the markers of choice. SSR are multi-allelic in contrast to next generation high-throughput sequencing (NGS) derived SNP marker which are bi-allelic. This makes SSR markers highly variable and useful for distinguishing even between closely related populations or varieties [21]. Furthermore, SSR are easily detected using standard PCR methods and are transferable to related taxa [22]. The development of NGS has recently enabled the identification of a large set of set of SSR sequences from sainfoin (Mora-Ortiz et al. 2016, BMC Genomics, accepted).

In this study, our aim was to develop and characterize a comprehensive set of markers based on recently identified SSR sequences (Mora-Ortiz et al. 2016, BMC Genomics, accepted) in a panel of 32 sainfoin individuals of different origin.

## Methods

### Plant material

In order to include a large range of genetic diversity, we selected a set of 32 individual sainfoin plants from 29 different accessions (Table 1), originating from a range of geographical regions and showing differences for tannin content and composition [12, 13, 23, 24]. These accessions were grown in the glasshouse at the National Institute of Agricultural Botany (NIAB) (Cambridge UK) and in the field at Agroscope (Zurich, Switzerland). Young leaf material was collected from each single plant, ground in liquid nitrogen and stored at −80°C until subsequent DNA extraction.

**Table 1:** *O. viciifolia* individuals used for marker characterization in this study

Individual number	Variety	Status	Origin	Source
ID_01	247	NA	Morocco	GRIN
ID_02	Buceanskij	NA	Romania	GRIN
ID_03	CPI 63750	NA	Turkey	GRIN
ID_04	CPI 63764	wild	Turkey	GRIN
ID_05	CPI 63820	NA	Spain	GRIN
ID_06	CPI 63826	NA	Spain	GRIN
ID_07	NA / RCAT028437	NA	Hungary	GRIN
ID_08	Ökotyp Wiedlisbach	ecotype	Switzerland	ISS
ID_09	Premier	landrace	Switzerland	ISS
ID_10	Rees A	cultivar	UK	GRIN
ID_11	TU86-43-03	cultivated	Turkey	GRIN
ID_12	Nova	cultivar	Canada	GRIN
ID_13	Visnovsky	cultivar	Czech Republik	ISS
ID_14	Perly	cultivar	Switzerland	ISS
ID_15	Brunner	landrace	Austria	ISS
ID_16	Perdix	cultivar	Switzerland	ISS
ID_17	Cotswold Common	cultivar	UK	RAU
ID_18	Perly	cultivar	Switzerland	RAU
ID_19	Somborne	cultivar	UK	RAU
ID_20	Ibaneti/ RCAT028292	NA	Romania	RCAH
ID_21	Bivolari/RCAT028294	cultivar	Romania	RCAH
ID_22	NA/170582	NA	Hungary	RCAH
ID_23	CPI 637554/ 192995	NA	Turkey	GRIN
ID_24	CPI 63767 / 212241	cultivar	USA	GRIN
ID_25	Na/228352	wild	Iran	GRIN
ID_26	CPI 63781/ 236486	NA	Turkey	GRIN
ID_27	Cholderton Hamshire Common	cultivar	UK	GRIN
ID_28	Visnovsky	cultivar	Czech Republic	GRIN
ID_29	Zeus	cultivar	Italy	Cotswold Seeds Ltd
ID_30	Zeus	cultivar	Italy	Cotswold Seeds Ltd
ID_31	Ambra	cultivar	Italy	private
ID_32	Esparcette	cultivar	UK	private

*RAU* Royal Agricultural University Gloucestershire UK, *RCAH* Research Centre for Agrobotany Tápiószele; Hungary, *GRIN* Germplasm Resources Information Network, Washington, USA, *ISS* Agroscope Institute for sustainability science, Zurich, Switzerland

### DNA extraction

DNA was extracted using the Nucleon Phytopure Genomic DNA extraction kit (GE Healthcare, Little Chalfont Buckinghamshire, United Kingdom) following the manufacturer’s instructions. This method has been shown to be suitable for extraction of high quality DNA from *O. viciifolia*, in which high levels of polyphenol and condensed tannins have been reported to interfere with a successful DNA extraction using other approaches [14]. DNA quality and quantity was assessed using gel electrophoresis and spectrophotometry.

### PCR and Gel electrophoresis

A total of 400 SSR primers designed from *O. viciifolia* transcriptome data (Mora-Ortiz 2016, unpublished), were tested with unlabeled primers for amplification in the 32 plants using an iCyler (Biorad, Hercules, USA) in a volume of 10 μL, with 10 ng DNA, 1 x Go Taqflexi buffer (Promega, Madison, USA), 2.5 mM MgCl_2_ (Promega), 0.2 mM dNTPs (Promega), 0.2 μM forward primer, 0.2 μM reverse primer and 0.5 U Polymerase G2 (Promega). The conditions followed a touchdown PCR approach with 4 min at 94 °C, 12 cycles of 30 s at 66 °C with −1 °C decrease at each cycle plus 30 s at 72 °C, and 30 cycles of 30 s at 94 °C, 30 s at 54 °C plus 30 s at 72 °C, followed by 7 min at 72 °C. PCR products were separated by gel electrophoresis. Amplicons were separated on 1 % agarose in 1x TBE buffer, stained with ethidium bromide and visualized under UV light.

### M13 PCR and capillary electrophoresis

Those 101 primer pairs that successfully amplified fragments in the 32 individuals (Table 2) were further characterized for polymorphisms using the M13 (–21) tail primer genotyping protocol [25]. The PCR reactions were conducted in an iCycler (Biorad) in a sample volume of 10 μL, each containing 20 ng DNA template, 1x Go Taqflexi buffer (Promega), 1.5 mM MgCl_2_, (Promega), 0.2 mM dNTPs (Promega), 0.16 μM forward primer carrying the M13-tail, 0.04 μM reverse primer and 0.16 μM fluorescently labelled M13-primer, 0.5 U polymerase GoTaq G2 (Promega).

**Table 2:** SSR primer sequences used for amplification in 32 *O. viciifolia* individuals and characteristics of SSR motifs

Marker	Motif	Repeats	Predicted size	Forward primer (5′–3′)	Reverse primer (5′–3′)
OVK002	AG	9	164	CCCACCAGACAAAAAGAATA	GCTTTCCCCTTCATCAACTAT
OVK003	TA	8	122	GATAGAATTCGTTTGTTGGTG	ATCTTTGTAACTGTTCGCTCA
OVK017	AC	8	158	GGGTGTTAGTTATCCATTTCC	ACATACTAGCCTTCTGGGGTA
OVK027	CTCG	6	129	AATGGAATCTCGGAGACAG	GGAAGAAGACGAAGTAGTAGGA
OVK034	GCT	6	150	GTGAGATGAGCTTGGACATT	AGATAACTAACTGCAGGCAAG
OVK036	AGGT	6	150	GTGTTAAAGGGGTGAAAACAT	CATTTTGACAAACCAGTATCC
OVK038	ATT	6	166	CCACATACGAGACAGAATAGG	CTGAAAATTGATCGATACTGG
OVK042	GTT	6	144	GGAACGGTTAATTTCTGATTT	AGAATTCCGTACAAGTCGAG
OVK045	AGA	6	148	CCAAAAATCATCAATCAACAC	TTGAACAAGGGTTAGGGTTAT
OVK046	AGTG	6	151	TCAACCACATTATAAAACCTCA	CGCGAAATCATAGTTCACTT
OVK054	GAA	6	201	TTGCAGAGATAACACTCACCT	TCCTGAAAAACCTAATCACAA
OVK055	GAT	6	189	GAAGATATTTCAAAGCAGCAA	CATGCTACCACTAGCAGAAGT
OVK063	TTG	6	188	AATTGCAACTGAAACTGAAAC	ACTGCTACCCTCTCCATAAAT
OVK068	GGA	6	195	GACCACCCGCAGCTCAAC	GTCTTCTTCCCCCATATTTAG
OVK072	ACC	7	199	TTGCCTTAGTCAGTTACCTTG	GTGGAGAGAATGAGAGAACCT
OVK073	GAC	6	200	GTAGACAACCGTATCTGGACA	AAGATGGAAGGTTCTAGTTCG
OVK077	TTA	6	249	GTCCCTCTCTCTCAAATTGTT	AGGTTAATGGAGCTTAGTGGT
OVK089	CAT	6	257	CAAAGTCATACCAATCACCAT	TCTTGGAAGCACTTGTTACTC
OVK093	CCA	6	259	CCAAGTGTTTGAAAGTCTCAG	TGAGAGTTCGTTCAAGGTAGA
OVK094	TTGCG	5	255	ACCGATCTTAGGATAGATGGA	ACTTTTGGTTGCTTAGTCGAT
OVK096	TCA	6	249	GAGCGTTGCATTTACATTTAC	CATCCTCCTTTACACCCTAAT
OVK097	GTGA	6	252	TCTATAGAGATGAGGCGACAA	CGCCCCTAACTAACCTACTAC
OVK099	TGAG	6	247	AGAAAATGGAAGCAACAGAGT	ACAAATAGCAGCTCCCTTC
OVK101	CTAA	6	254	GTTGAGTTTCAGACACAGAGC	AATAGCTCCCACAATAACTCC
OVK102	TGT	6	249	CCAAAGGGTGTTTTATTTTCT	GGAAGAAATTAAGCAAATGGT
OVK107	AG	8	193	AAGTTAAAACTTTGCGTTGTG	GACGTTGTTCTGGATTTCTTC
OVK111	GGT	7	206	TATAGACCTTCTCCTCCCAGA	GTGAAAGTCACAAATCCAAAG
OVK119	CAG	6	199	ACCCTCCTTCTCTCCTTATTT	GACGAGAGAACTCGTTTATGA
OVK122	TC	9	211	GCAGATAGCACAGTTATCGAC	GAACCACACACACAGAATCA
OVK123	ACA	9	200	CACCCATTAACTATCATGGTC	CAAGCCCTTTGTGAGATACTA
OVK124	TGA	9	211	GCCTTTTCTGTGACTCGTAA	GCTCCATTCCCATTTATAGTT
OVK125	CATTT	5	193	AAATTTAAGCACCGGAATAAC	AAAGCAAAAGGGCTACTAAAG
OVK126	TC	8	197	CGACAAAACTATTTAGGCAAA	GGGAAGAGATCATAAACCCTA
OVK127	AT	8	200	GCCCAAAATGTATTATCCTTC	AGAACAGACAGATATGCAAGC
OVK131	TA	8	200	TCTATCTGGGTGTTGTTTTGT	CTGTTTGAATATCGATTACCAC
OVK133	TG	8	196	TGCTTCAGCATTATTGTAACAT	TGCACTTCTCCATACTTCCTA
OVK138	CTAA	6	250	TAATATGGTGCAAGTTCCAAT	TTCTACGCTTAGCTCAAACC
OVK141	CACG	6	239	GAGGAGGTACATACAGCACAG	CAACCTCCTCGTTATCTTTTT
OVK142	GT	8	243	AACATGACTACTGTGAACAAGG	CGAACATGTAATTGATCCAAG
OVK155	GTG	6	251	CAGGTTTGAAGTAGCAGAGAA	GTAGACCACGCATACTGAATC
OVK158	GACT	6	257	TCAGAGTGTGTTGTGTTGTGT	AGTGAAGCAAATGTGTGATTT
OVK159	TG	8	251	CATTATTGCCTAGCATTGTTC	ATTTCACCATCAAGTATGCAC
OVK161	TTCC	6	249	AAAGCTTTCTACACGTTGGTA	TGGGTTTTTACACTCTGTGAT
OVK165	ACA	6	267	TTTCAAACACTCACTCACTCC	TCGGATTTGTGACCTAACTC
OVK168	TGA	6	253	AATTATCACCCACTGCTATGA	GGTTTCCATCACTGTTTGTTA
OVK172	AGC	6	256	TTATTAAACCTGCGTCTTCTG	GTAGAGCTGTGGGCTTTATCT
OVK173	CT	8	253	TCGTTCTCGTGATTATTCTGT	CCTCTATTCAAATAGGGCAAT
OVK174	GGCCC	5	246	ACATGATCGTGAATATGAAGC	CAGCAGCAATCAATATATCATC
OVK175	CA	9	250	GTAAAATATCAAGCAGGAGCA	AAACTATGCAGACACCCTGTA
OVK177	CTG	7	257	TCTGTTGATTTAAGGAGACGA	CTCTTGCTCATATTTTCCTCA
OVK181	AAG	6	257	AGGAAGAAGAAGAAGAAGCAG	TTCTCCTTTAACCACAACCTT
OVK183	TGAT	6	256	GAGGGTAAGAGAGAGTGGAAG	CTTGCCTGATATCTTCTCAAA
OVK196	AGC	6	286	TTTTGAGAGTGTGGAAGGTTA	AGTATGAGCCTGATGATGATG
OVM003	TC	9	297	CCGTCTGTTTAATCATTCACT	GAAAGGAAAGGTTATTGGAGA
OVM004	ATTT	5	290	GGGAATTCTTAAATCTCATGG	ATGCATGGTACTGGGTCGT
OVM025	CAA	6	297	TTCTGAACAACAACAACAACA	GTCCAGGAGCTAAGTAACCAT
OVM031	TGA	6	306	ATTGGTTTCTAAGGAGGACTG	GCAATACTCCTCTGCCTAGTT
OVM033	CTC	6	300	CAAGGCTTATTTGGTTAACAG	ATACTATTTCCCATGCCTACC
OVM034	TTC	6	308	GCATTTCATCAAACACTTTTC	TTGGTTTGAATCTGTGAGACT
OVM035	TTC	7	303	TCATCAAACACTTTTCGTTCT	TTGGTTTGAATCTGTGAGACT
OVM038	GAAG	6	297	CACAGGACAAGAGTGAGAGAG	TCATGATACCACGAATTTTTC
OVM043	GAG	7	167	TAGTATGGCTGAAATCAAAGG	ATATCATAAGGGCAACAGTGA
OVM048	AT	9	157	GACATTGAAATCAAACAATCC	AACACTTGTCATGTTTCCAAG
OVM049	TGA	7	150	AACAAACAAGAGGAAAAGGAG	TATGTGCTTATCAGGCATTTT
OVM050	ATCC	6	161	ATGAGCATGAAGAGTTTCAGA	ACACATCTACGACTTCTTTCG
OVM053	GTGGA	5	149	CACCAAAAGCATAGCAATAGT	GCTTGAATTGAATGAGAAATG
OVM057	TTG	6	153	CCTTGAGGAGGAATAATAGGA	GACATCATCATCACCTTCACT
OVM058	AT	9	150	GTCAAGTCATACCCATACGAG	CAGTGTAACCATATGCACAAA
OVM059	AGA	6	149	ACTCCAACTCCAACTCAGAAC	AAGCGAAGAAGAGAGTGAGAT
OVM060	CT	8	159	ATGTAATCAAAAGGTGCAGAA	AGCTTCCAAAACAGTGTATGA
OVM061	GTA	6	150	TTAACACACGTACGTACCACA	TTTGTCGTTGATCGTTAAGTT
OVM062	AG	8	139	GGAAAAAGGTTTGGATAGATG	AAGTTTTCCCCACACTATTCT
OVM064	AT	8	353	GCATGCACAGAATTAAGTTTC	AGAAGGTCCTTTGAAAATCAG
OVM065	CT	8	352	AAGACAGCGAGTTACCAATCT	GATTGAAACTGAGTAGCGATG
OVM067	CTT	6	352	CAACCTTAATACCAACCTTCC	AAAAGTAGCCAGAGAGCAAAT
OVM068	CCT	6	333	CTACAACTCACCGAAACTCAC	CGATTTCTGCCTCTTTATTCT
OVM069	AATG	6	357	ATGTTGTACAGATGAGCTTCG	TAGTGAGCAAACCTATTTTGG
OVM072	GAA	6	350	TTGATGTGGTTGATCCTATTC	GATGTCAACATCTTGGTCATTA
OVM073	ACA	6	346	GTTCTCAAACGCACTATCAAC	AAAATCTTGTAGGGATTCGAT
OVM076	AAC	7	348	CCCATTCTTCATCTTTCTCTC	TGCTTCCATAATCAGTGAAAT
OVM081	GT	9	350	TCTAGCACAATGTTTTGGATT	TATTGAGTTGAAGCAGACCAT
OVM083	CT	8	347	CACACAAACACAAAACTCACA	GATCGGAGAAAAGAAGAAGAG
OVM086	GAA	6	350	TCATACAAAGTTCCTTCCGTA	ATTGCCAATAACAGTGAAGAG
OVM090	CCA	6	151	AATCAATGGAGGAGGATAAAC	GAAGGTTGAAAAGGGAATAAA
OVM091	ATC	6	188	AACCACCCTTAATTCCATAAG	AGATAAAAGCCGCAAAAGTAT
OVM092	CAC	6	157	GGACCAACAAAGAGGATTATT	CCCTTGCTTGAAGTGTTACTA
OVM094	GTTT	5	163	ATTCATGGGGACAATAAATTC	CAAGAGAATGAATGAATCAGC
OVM099	GA	9	149	TATGTATTGCAGAATCACAGC	TATTACCCTTTTCCATCTTCC
OVM100	AAG	6	151	GAACTAGATTTGCGGCATT	CCCACACCCTTATCCTTATTA
OVM110	AT	8	154	CTGGACGAAAACAACATATTC	GTTGGCTTTGGTACTGACATA
OVM116	GAT	7	151	AACTACACGCACGTAATGAAT	TGGTTTGATAAACACCTCAAG
OVM120	TTC	6	152	TTCAGTGTCACTTTCCTCATT	AGAAGTTGTCATGTCAAGGAA
OVM122	TGG	6	156	ATGAATCTTGTACGGAATCTG	GAAGAAAAAGCCATAAACACC
OVM125	AAATT	5	151	ATTCTTTCAACAAGCAAGTGA	CTGCAATTCCATCCTATTTTA
OVM126	TCC	6	188	ACTAAGAACCACCCAAAACAT	TGAGAAGATGGAGAAGATGTG
OVM128	TGTT	6	155	GAGAAGCATAACCAAAATCCT	TGGAAGAAAAGAAACTTCTGA
OVM129	TG	8	133	AATTGGATTCATGTGTTAGGA	GAAGTGGAGCCAAAACCT
OVM130	AG	9	154	GCAAATTATCACCATGCAC	CGTGAAGAAAATCGGTACTTA
OVM131	AGA	6	153	GAAATAACGCAGGCAGATAC	AATTAGAGGCTTCGACTTGTT
OVM132	GAC	6	142	ACGGTAATCAGTAGTGACAGC	GTGTGACAGAAAATGGGATTA
OVM133	TTTC	5	171	TAGCATCAAGGTTGGAAATAG	CTAGGCTACCTGAATCAAACA

PCR conditions were 2 min at 94 °C, 30 cycles of 30 s at 94 °C, 45 s at 56 °C and 45 s at 72 °C, followed by 8 cycles of 30 s at 94 °C, 45 s at 53 °C and 45 s at 72 °C. The final extension step was conducted at 72 °C for 10 min. An aliquot of 1μl of the PCR product was diluted in 10 μl HiDi™ formamide (Applied Biosystems®, Thermo Fisher Scientific, Waltham, MA, USA) and 0.2 μl Rox 500™ oligonucleotide ‘size ladder’(Applied Biosystems®) for capillary electrophoresis on the Genetic Analyzer 3730 (Life Technologies, Carlsbad, CA). Alleles were scored using the GeneMarker software (Softgenetics, V2.4.0 Inc., State College, USA).

### Data analysis

All statistical analyses and calculations were performed using R statistical software (R Core Team, 2014). The polymorphism information content (PIC) of SSR markers was calculated as the mean of the PIC of each allele, using the formula for dominant markers from Roldan-Ruiz et al. [26] as;

$$ \mathrm{PI}{\mathrm{C}}_{\mathrm{i}} = 2{\mathrm{f}}_{\mathrm{i}}\left(1\ \hbox{-}\ {\mathrm{f}}_{\mathrm{i}}\right), $$

where PIC_i_ is the polymorphism information content of allele i and f_i_ is the frequency of occurrence of allele i (fragment present) in the 32 individuals. From single alleles, average (PIC_Av_), minimum (PIC_Min_) and maximum (PIC_Max_) PIC values were calculated for each SSR marker.

In order to calculate genetic distance measures, SSR alleles were coded as individual markers with 1 for presence and 0 for absence of the allele as binary data. Pairwise genetic distances between individuals were calculated as modified Rogers’ distance D_w_, [27] which shows the extent of genetic diversity between two individuals [28] ranging from 0 (no diversity between individuals) and 1 (maximum diversity).

Genetic relationships were visualised using cluster analysis and the R-function pvclust() [29] based on Euclidean distance that was rescaled to D_w_ for plotting purposes (D_w_ and Euclidean distance show a linear relationship, Additional file 1: Figure S1). Probability values (*p*-values) were calculated for each cluster using multiscale bootstrap resampling [30, 31] to calculate approximately unbiased (AU) *p*-values [32]. The k-means clustering algorithm [33] was applied to the D_w_ values using a sequence of k = 2 clusters to 32 clusters. The Calinsky criterion [34] was then calculated for each number of k as implemented in the R function cascadeKM() and the optimum number of clusters was determined at the maximal value. Population structure was further investigated by principal component analysis performed on binary raw data of individual alleles.

## Results

### SSR analysis

SSR markers showed a high degree of polymorphism and overall, 1154 alleles were found with an average of 11.4 alleles per marker locus (Table 3). Among those 1154 alleles, only five alleles (from SSR OVK042, OVK172, OVM031, OVM072 and OVM100) were non-polymorphic and hence present in all individuals studied.

**Table 3:** Characterization of the 101 polymorphic sainfoin markers

Marker	PIC Av	PIC Min	PIC Max	NoA	NoA Priv	MinAF	MaxAF	Size
OVK002	0.22	0.06	0.47	9	3	0.03	0.63	154–175
OVK003	0.23	0.06	0.47	11	2	0.03	0.38	92–124
OVK017	0.22	0.06	0.50	19	5	0.03	0.47	148–184
OVK027	0.28	0.06	0.50	9	2	0.03	0.59	120–140
OVK034	0.27	0.06	0.49	12	1	0.03	0.56	138–154
OVK036	0.35	0.17	0.50	7	0	0.09	0.69	133–154
OVK038	0.19	0.06	0.40	14	4	0.03	0.28	155–186
OVK042	0.25	0.00	0.50	2	0	0.50	1.00	183–186
OVK045	0.29	0.12	0.43	6	0	0.09	0.94	138–148
OVK046	0.31	0.06	0.49	12	1	0.03	0.56	138–157
OVK054	0.29	0.12	0.49	15	0	0.06	0.44	274–290
OVK055	0.20	0.06	0.38	8	2	0.03	0.84	135–159
OVK063	0.24	0.06	0.50	13	2	0.03	0.72	179–200
OVK068	0.25	0.06	0.43	9	3	0.03	0.31	186–213
OVK072	0.32	0.12	0.50	4	0	0.06	0.81	193–198
OVK073	0.29	0.06	0.50	11	1	0.03	0.53	186–210
OVK077	0.23	0.06	0.45	9	2	0.03	0.78	233–264
OVK089	0.27	0.06	0.49	9	2	0.03	0.44	279–299
OVK093	0.23	0.06	0.50	14	6	0.03	0.56	234–271
OVK094	0.24	0.06	0.48	14	4	0.03	0.66	208–244
OVK096	0.21	0.06	0.48	20	6	0.03	0.41	215–294
OVK097	0.22	0.06	0.38	3	0	0.13	0.97	240–248
OVK099	0.25	0.06	0.49	13	2	0.03	0.75	232–270
OVK101	0.36	0.06	0.50	7	1	0.03	0.72	339–352
OVK102	0.23	0.06	0.34	4	1	0.03	0.22	239–251
OVK107	0.29	0.06	0.45	15	1	0.03	0.72	206–234
OVK111	0.26	0.06	0.48	7	2	0.03	0.75	213–232
OVK119	0.30	0.06	0.47	10	1	0.03	0.72	216–252
OVK122	0.24	0.06	0.45	8	1	0.03	0.66	330–341
OVK123	0.26	0.06	0.50	10	3	0.03	0.75	208–237
OVK124	0.26	0.06	0.49	15	1	0.03	0.44	218–267
OVK125	0.29	0.06	0.50	9	1	0.03	0.72	197–222
OVK126	0.25	0.06	0.49	15	3	0.03	0.56	198–233
OVK127	0.28	0.06	0.49	6	1	0.03	0.44	204–222
OVK131	0.17	0.06	0.48	15	3	0.03	0.59	183–228
OVK133	0.25	0.06	0.50	13	3	0.03	0.63	205–239
OVK138	0.21	0.06	0.49	13	6	0.03	0.56	232–267
OVK141	0.14	0.06	0.47	15	7	0.03	0.38	242–269
OVK142	0.25	0.06	0.50	12	3	0.03	0.47	256–285
OVK155	0.24	0.06	0.49	14	3	0.03	0.56	234–282
OVK158	0.19	0.06	0.40	24	6	0.03	0.28	273–375
OVK159	0.23	0.06	0.50	14	4	0.03	0.81	268–290
OVK161	0.25	0.06	0.40	12	1	0.03	0.28	220–276
OVK165	0.19	0.06	0.50	20	6	0.03	0.50	273–311
OVK168	0.24	0.06	0.50	11	3	0.03	0.81	258–284
OVK172	0.16	0.00	0.30	5	0	0.06	1.00	268–279
OVK173	0.23	0.06	0.48	18	5	0.03	0.59	268–316
OVK174	0.23	0.06	0.48	5	2	0.03	0.75	245–266
OVK175	0.19	0.06	0.38	10	2	0.03	0.88	252–267
OVK177	0.27	0.06	0.49	7	2	0.03	0.59	267–286
OVK181	0.19	0.06	0.38	19	4	0.03	0.25	343–381
OVK183	0.24	0.06	0.49	17	1	0.03	0.44	266–289
OVK196	0.21	0.06	0.50	8	2	0.03	0.53	297–314
OVM003	0.31	0.12	0.47	10	0	0.06	0.69	299–321
OVM004	0.21	0.06	0.45	18	6	0.03	0.34	380–426
OVM025	0.33	0.17	0.49	7	0	0.09	0.84	306–324
OVM031	0.26	0.00	0.50	13	1	0.03	1.00	292–353
OVM033	0.29	0.06	0.50	8	1	0.03	0.69	308–330
OVM034	0.22	0.06	0.50	17	5	0.03	0.53	307–355
OVM035	0.22	0.06	0.50	17	5	0.03	0.53	301–350
OVM038	0.19	0.06	0.43	14	4	0.03	0.31	311–351
OVM043	0.30	0.06	0.49	10	2	0.03	0.66	173–203
OVM048	0.29	0.12	0.43	6	0	0.06	0.72	174–186
OVM049	0.31	0.06	0.50	9	1	0.03	0.50	162–198
OVM050	0.20	0.06	0.49	13	6	0.03	0.72	168–198
OVM053	0.32	0.06	0.50	11	1	0.03	0.50	134–182
OVM057	0.35	0.17	0.49	5	0	0.09	0.66	165–180
OVM058	0.23	0.06	0.49	15	3	0.03	0.44	135–178
OVM059	0.24	0.06	0.48	7	2	0.03	0.59	156–174
OVM060	0.23	0.06	0.50	21	4	0.03	0.50	172–219
OVM061	0.19	0.06	0.50	10	4	0.03	0.84	143–175
OVM062	0.30	0.06	0.49	12	2	0.03	0.59	151–170
OVM064	0.16	0.06	0.47	16	8	0.03	0.38	380–444
OVM065	0.25	0.06	0.49	14	4	0.03	0.69	360–391
OVM067	0.33	0.17	0.49	6	0	0.09	0.66	366–380
OVM068	0.26	0.12	0.43	8	0	0.06	0.88	343–368
OVM069	0.26	0.06	0.48	13	2	0.03	0.59	454–479
OVM072	0.28	0.00	0.50	7	1	0.03	1.00	365–387
OVM073	0.20	0.06	0.45	21	5	0.03	0.34	446–511
OVM076	0.22	0.06	0.48	17	3	0.03	0.41	347–376
OVM081	0.18	0.06	0.40	17	5	0.03	0.28	353–396
OVM083	0.30	0.06	0.50	11	2	0.03	0.63	365–384
OVM086	0.32	0.06	0.50	10	1	0.03	0.63	371–391
OVM090	0.30	0.06	0.49	8	2	0.03	0.84	158–180
OVM091	0.34	0.06	0.50	8	1	0.03	0.47	184–217
OVM092	0.23	0.06	0.43	7	2	0.03	0.81	163–185
OVM094	0.33	0.12	0.50	7	0	0.06	0.81	190–207
OVM099	0.18	0.06	0.50	11	5	0.03	0.50	165–198
OVM100	0.24	0.00	0.48	5	0	0.09	1.00	163–179
OVM110	0.21	0.06	0.49	18	5	0.03	0.44	163–185
OVM116	0.28	0.06	0.49	15	2	0.03	0.56	138–204
OVM120	0.34	0.06	0.50	6	1	0.03	0.88	169–187
OVM122	0.31	0.06	0.50	4	1	0.03	0.91	164–180
OVM125	0.26	0.06	0.50	10	3	0.03	0.47	161–180
OVM126	0.22	0.06	0.50	18	4	0.03	0.53	191–229
OVM128	0.26	0.06	0.47	9	1	0.03	0.81	173–190
OVM129	0.25	0.06	0.49	14	4	0.03	0.56	146–173
OVM130	0.20	0.06	0.47	20	7	0.03	0.38	152–187
OVM131	0.34	0.06	0.50	8	2	0.03	0.56	159–198
OVM132	0.30	0.06	0.47	8	2	0.03	0.78	157–176
OVM133	0.20	0.06	0.47	14	3	0.03	0.63	177–212

PICAv, PICMin and PICMax give the average, minimum and maximum allele-wise polymorphism information content values, NoA_Tot_ the total number of alleles, NoA_Priv_ the number of private alleles, MinAF the minimum allele frequency and MaxAF the maximum allele frequency value

With only two alleles in the 32 individuals, SSR OVK042 had the lowest number of alleles, whereas OVK158 had the highest number with 24 amplified alleles. The minimum rate of allele occurrence was 0.03125, corresponding to occurrence in only one genotype (i.e. a private allele of an individual genotype). In total, 250 private alleles were detected and these were equally distributed across the examined set of individuals and markers. With regard to individuals, the highest number of private alleles over all markers was found for individual ID_08 (14 private alleles) and the lowest number was found for ID_17 (3 private alleles). The origin of the individual did not appear to affect the occurrence of private alleles. With regard to markers, the most private alleles were observed in OVM064 (8 private alleles), whereas 16 markers (15.8 %) had no private alleles at all.

The average polymorphism information content (PIC_Av_) ranged from 0.14 (OVK141) to 0.36 (OVK101) (Table 3). A detailed look at the PIC values of individual alleles in the different markers exhibited minimum PIC values per SSR (PIC_Min_) between 0 (Additional file 2: Figure S2), OVK042, OVK172, OVM031, OVM072, OVM100) and 0.17 (OVK131) and maximum PIC values per SSR (PIC_Max_) between 0.3 (OVK 172) and 0.5 (16 different markers).

The overall length of SSR fragments detected ranged from 91 to 511base pairs (bp). Markers with two base pair motifs had a slightly higher number of repeats (eight to nine) when compared to markers with three to five bp motifs (five to seven repetitions). The total fragment length observed did not differ between motif lengths (data not shown). Contrastingly, the number of alleles found for SSRs with two bp motifs was higher (13.5 alleles on average), compared to SSRs with longer motifs (10.7 alleles). The average number of alleles per sainfoin genotype was 230.1 over all SSR markers, leading to an average of 2.3 alleles per SSR marker and genotype. The lowest number of alleles was found for genotype ID_25 with 191 alleles, the highest for ID_07 with 268 alleles. Assigning all individuals to cultivars and non-cultivars (ecotypes, landraces and NA) resulted in 981 alleles for individuals from cultivars (57.7 alleles per individual) and 942 alleles for non-cultivars (62.8 alleles per individual).

### Diversity of O. viciifolia individuals

**Fig. 1:**
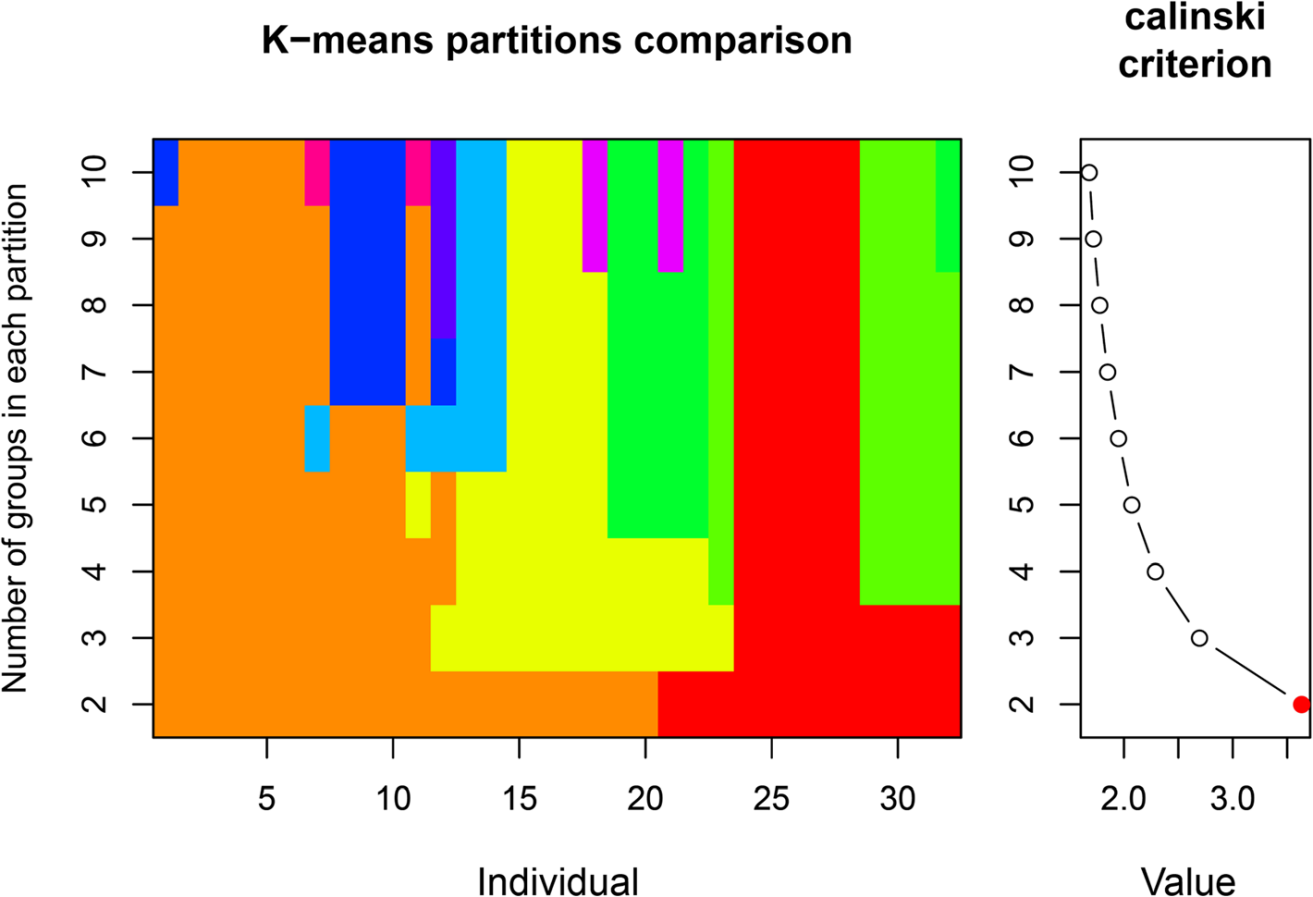
Group separation of individuals as assessed by k-means partitioning for k = 2 to 10 with colors indicating different groups (left). The optimum number of groups (k) according to maximum Calinski criterion was determined to be two (right)

The allocation of individuals to groups by overall similarity of alleles was assessed using k-means partition comparisons. Those k-means statistic (Fig. 1, left) simulate a grouping of individuals (assigned by different colors) dependent on number of groups chosen. Individuals were assigned into two to ten groups, with a more homogenous grouping for two and three groups. The Calinski criterion (Fig. 1, right), giving the most likely grouping by the highest value reached, indicating a grouping of individuals into two groups by a value >3.

**Fig. 2:**
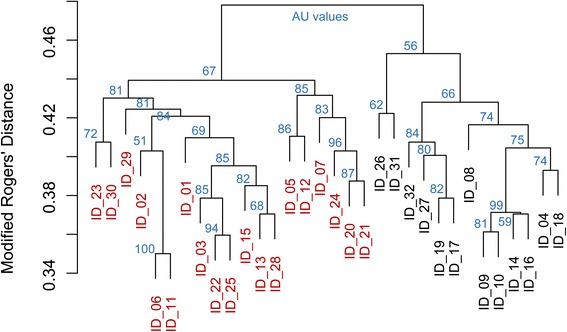
Cluster Dendrogram of individuals based on the modified Rogers’ distance. Values at branches are AU *p*-values (blue). Different colours of genotype labels give the affiliation to the two groups determined by k-means partitioning

The cluster dendrogram based on the modified Roger’s distance (Fig. 2) also displayed a partitioning of individuals in two main groups, which were separated by a modified Roger’s distance value of 0.47. Individuals belonging to the same variety located in the same main branch for the varieties Perly (ID_14, ID_18; 0.4), Visnovsky (ID_13, ID_28; 0.39) and Zeus (ID_29, ID_30; 0.48). The variety Perdix is an advanced variety originating from the variety Perly and the Perdix genotype (ID_16) clusters closely to one of the Perly individuals (ID_14).

**Fig. 3:**
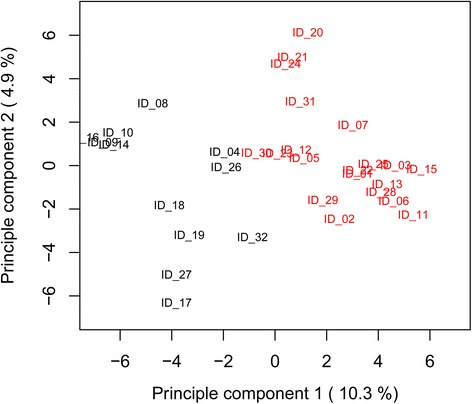
Principle component analysis of 32 sainfoin (*O. viciifolia)* individuals based on 1054 alleles of 101 SSR markers. Different colors give the affiliation to the two groups as determined by k-means partitioning

The first, smaller branch of the cluster (Fig. 2, right hand side) consisted mainly of individuals originating from Switzerland and the United Kingdom (cluster 1), whereas the majority of the second, larger branch was comprised of individuals from Southern and Eastern Europe as well as individuals from USA, Morocco and Canada (cluster 2). However, AU values showed no significance (values <95) for most branches. Principal component analysis (PCA; Fig. 3) showed a pattern comparable to that observed from cluster analysis with individuals of the two main clusters mainly being separated by the first principle component which explained 10.3 % of the total marker variation. The second principle component accounted for 4.9 % of the variation, most of which was intragroup. The occurrence of alleles across all markers varied between the two clusters with 849 alleles amplified in cluster 1 (65.3 per individual) and 979 alleles in cluster 2 (51.5 per individual).

## Discussion

The 101 SSR markers newly developed from sainfoin revealed a high degree of polymorphism. In addition to differences in multiples of the repeat motif, we also found alleles differing by fractions of the multiple motif length. Such variations could have arisen from insertions, deletions and translocations in the flanking region of the SSR [35]. Such mutations in the flanking region might also contribute to the high degree of polymorphism in our marker data set. The SSR sizes predicted through sequencing and the actual size distribution observed in the 32 individuals was consistent for most of the markers. Discrepancies can largely be explained by the fact that SSR motifs were developed from individuals not represented in the present study. In total, we found 1154 alleles at 101 loci resulting in 11.4 amplified alleles per SSR on average. This is twice the amount found by Demdoum [12], who found 5.83 alleles by transferring markers from barrel clover (*Medicago truncatula* Gaertn.) and soybean (*Glycine max* L.) to sainfoin. Fragments were smaller for the specific marker set in this study (92 to 511 bp) compared to markers adopted from other species [12] (79 to 865 bp). The larger sizes of alleles from cross-species amplification could be attributed to interspecific differences to the donor species due to repeat length variation within the SSR region and indels in the flanking region [36]. Avci [37] amplified 725 alleles from 18 SSR markers in diverse *Onobrychis* spp. using markers from pea and barrel clover. The higher number found by these authors could be explained by the larger diversity of germplasm used, which originated from different subspecies.

SSR marker studies with other tetraploid species using diverse panels of individuals showed lower numbers of alleles per marker compared to the present study, e.g. 7.2 alleles in sugar cane (*Saccharum officinarum*) [38], 6.7 alleles in switchgrass (*Panicum virgatum*) [39] and 6 alleles in peanut (*Arachis hypogaea*) [40].

A few markers were observed with less than five alleles among the 32 individuals. These may still be useful in future studies, since this study represents an initial screening of single individuals and not an extensive population survey. Additionally, using only the most polymorphic markers would bias the overall genetic diversity e.g. in conservation studies [41].

The challenge in analyzing SSR alleles in tetraploids lies in determining the dosage of each allele, which is often impossible using capillary electrophoresis for individuals carrying less than four different alleles at a specific marker locus. The PIC content gives an estimation of the information content of a marker and is traditionally calculated by the formula of Botstein [42]. This was developed for diploid species, for which the allele frequency is either known or can be inferred from the allele occurrence (presence/absence). For tetraploid species, the allele frequency is difficult to derive from the allele occurrence due to different allele doses (1 to 4 alleles). Hence, the formula for diploids could not be used for tetraploid sainfoin. Thus, the PIC was calculated separately for each allele, on the basis of allele occurrence counts, using a formula adopted from Roldan-Ruiz [26] and averaging the PIC across all loci of one locus [43]. Here, the maximum value that can be reached is 0.5, which corresponds to alleles found in 50 % of the population. Small values, on the other hand, correspond to very abundant or to very rare alleles. Deciding whether a SSR marker is useful also depends upon the scientific issue. Taking into account different allele-based PIC values of an SSR locus (Additional file 2: Figure S2), therefore, gives the most holistic picture of the SSR marker. High PIC values of alleles (0.5–0.4) are useful for inside population studies e.g. to trace marker trait associations, whereas low PIC values (0.0–0.1) of single alleles could be more useful for studies of evolution or genetic drift [44]. The average PIC values in this study indicated that most markers had alleles which could be found in a group of individuals and are suitable for several approaches in future studies. These PIC values were comparable to those found by Tehrani [43] which were between 0.16 and 0.44 in *Lolium persicum* Boiss. The large number of private alleles is a clear indication of genetic distinctness of the individuals, which was anticipated in view of their diverse origins.

Genetic diversity is a prerequisite for selection in variety development. So far, there is limited information on the genetic diversity of sainfoin available. Use of AFLP and SSR markers from other species were not able to reveal genetic diversity in distinct Spanish sainfoin accessions [12, 45]. The values of that study, given by Nei’s similarity values, which represent the proportion of shared fragments on the basis of binary data and corrected by the marker number [46], reached values of 0.73 to 0.8 [12, 45]. A conversion of those values to genetic distance values by the formula –ln (Nei’s similarity values) resulted in Nei’s genetic distance values of 0.31 and 0.22 [47]. In a study of sainfoin genetic diversity using RAPD markers in ten landraces from East Azerbaijan and in 36 Iranian sainfoin populations, Nei’s genetic distance values of 0.32 and 0.25, respectively, were observed [48, 49]. In our study, highest modified Roger’s distance of 0.48 corresponds to alleles not shared between our two cluster groups, which is almost 50 % (Fig. 2). The smallest Roger’s distance values with 0.35, corresponds to an approximate Nei’s distance value of 0.43 (Additional file 1: Figure S1), which is higher than the low values observed in other studies [12, 45, 48]. The majority of among-genotype comparisons showed higher values. The higher values of genetic diversity found in the present study may reflect the high variability of the markers developed and the selection of 32 individuals of contrasting origin. Despite the fact that individuals of the same cultivars in this predominantly outbreeding species can show considerable variability [50], the individuals from the same cultivar grouped clearly together in the present study (Figs. 2 and 3, Table 1).

The 32 individuals investigated separated into two clear groups based on different multivariate analyses. The first main group was comprised mainly of individuals from Switzerland and the United Kingdom, whereas the second group contained individuals originating from South and East Europe as well as USA, Canada and Morocco. In some instances, individuals originating from the same geographical region did not cluster tightly together, some even into the two different cluster groups. The three plants from Italy, ID_29 and ID_30, both cultivar “Zeus”, clustered in group 2, whereas ID_31 of the cultivar “Ambra” clustered to group 1). Especially for cultivars, this is likely to be due to different origin of base material (which is often unknown), as well as divergent breeding and selection history.

A similar grouping of accessions identified by the present cluster analysis could be found in earlier studies between sainfoin accessions from Western Europe and those from Eastern Europe and Asia [12, 23]. This clear genetic distinction between the individuals from Western Europe and those from Eastern Europe and beyond could reflect adaptation to diverse climatic conditions either naturally or as a result of local selection by growers [44]. Under genetic isolation and limited gene exchange, differentiation in the sainfoin germplasm with accompanied morphological separation seems likely [51]. The average number of alleles amplified in individuals of the West European cluster was 65.3, which was approximately 14 alleles more than individuals from the other cluster (51.5). These results might indicate a higher allelic diversity in individuals from mainly Switzerland and Great Britain compared to other origins. Deducing differences in tannin content and composition between single individuals of the two clusters based on earlier studies dealing with samples of plants from the same accessions is extremely difficult because the variation found within accessions is at least as large as variation between accessions [24].

## Conclusions

This study reports the first characterization of specific co-dominant SSR markers for sainfoin. The 101 SSR markers characterized in this study showed a high degree of polymorphism and clearly demonstrated the differences between sainfoin individuals, with diverse origin, on a molecular genetic level. The genetic differences found in our panel separated the individuals into two groups, with a clear correlation to the geographical origin of those individuals. SSR markers, such as those characterized here, will be very useful in future genetic analyses, such as paternity or pedigree analysis in breeding programs, as well as more detailed analysis of genetic diversity in this forage crop. Furthermore, the development of new varieties could be crucially improved by choosing distinct genepools and minimising inbreeding depression.

## Additional files

Additional file 1: Figure S1. Relationship between modified Roger’s Distance to Euclidian Distance and to Nei’s Distance. (PDF 319 kb) Additional file 2: Figure S2. Polymorphism Information Content (PIC) values for individual alleles at SSR loci. Different grey levels are used for better visual differentiation among alleles of the different SSR markers. (PDF 431 kb)

## Abbreviations

AFLP: Amplified fragment length polymorphism
EST-SSR: Expressed sequence tag – short sequence repeats
ITS: Internal transcribed spacer
RAPD: Random amplified polymorphic DNA
SNP: Single nucleotide polymorphism
SRAP: Sequence related amplified polymorphism
SSR: Short sequence repeats
