# Supplementary material for: Characterization of novel SSR markers in diverse sainfoin (Onobrychis viciifolia) germplasm
Source: BMC Genet. 2016 Aug 30;17(1):124. doi: 10.1186/s12863-016-0431-0 (PMC5006395; doi:10.1186/s12863-016-0431-0)

Additional file 1: Figure S1

Figure S1 Relationship between modified Roger’s Distance to Euclidian Distance and to Nei’s Distance.

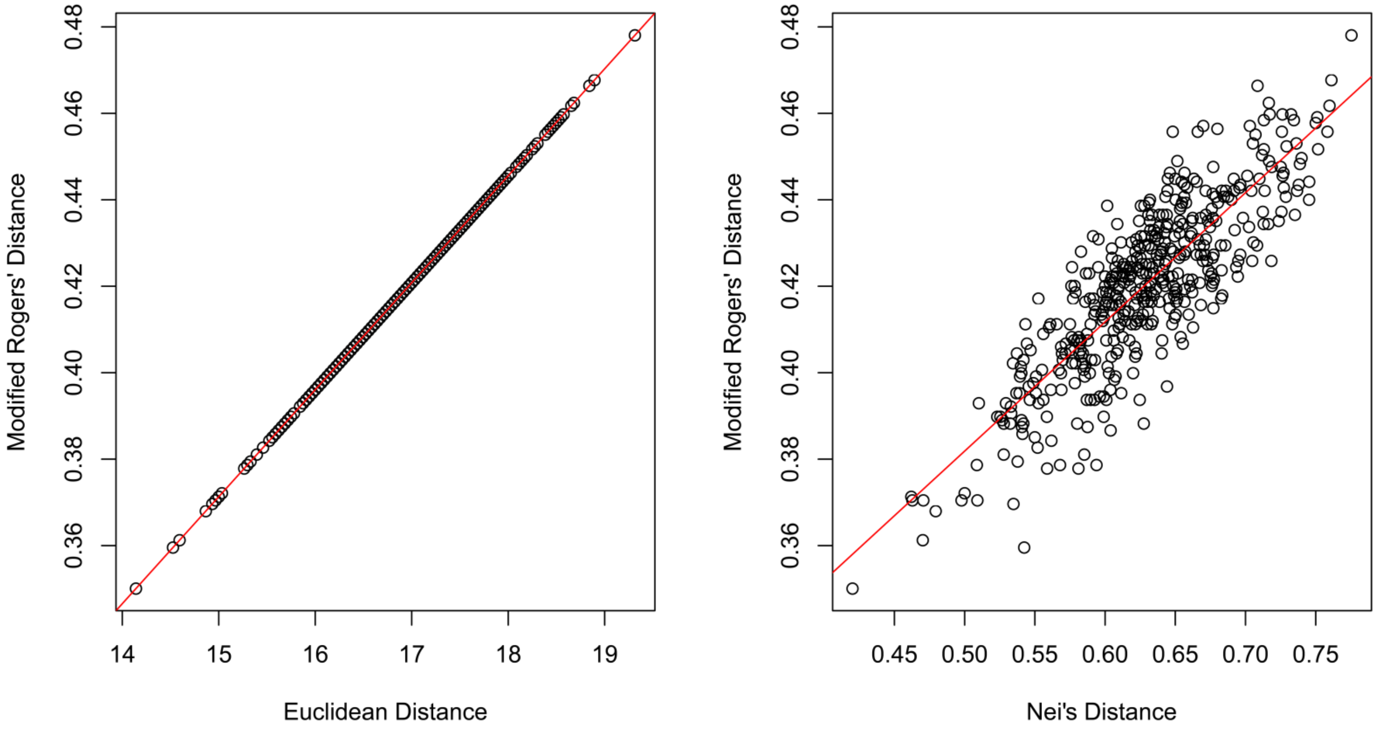

Supplement: Additional file 1: Figure S1. — Relationship between modified Roger’s Distance to Euclidian Distance and to Nei’s Distance. (PDF 319 kb) [file 12863_2016_431_MOESM1_ESM.pdf]
