# Supplementary material for: Characterization of novel SSR markers in diverse sainfoin (Onobrychis viciifolia) germplasm
Source: BMC Genet. 2016 Aug 30;17(1):124. doi: 10.1186/s12863-016-0431-0 (PMC5006395; doi:10.1186/s12863-016-0431-0)

Additional file 2: Figure S2

Figure S2 Polymorphism Information Content (PIC) values for individual alleles at SSR loci. Different grey levels are used for better visual differentiation among alleles of the different SSR markers.

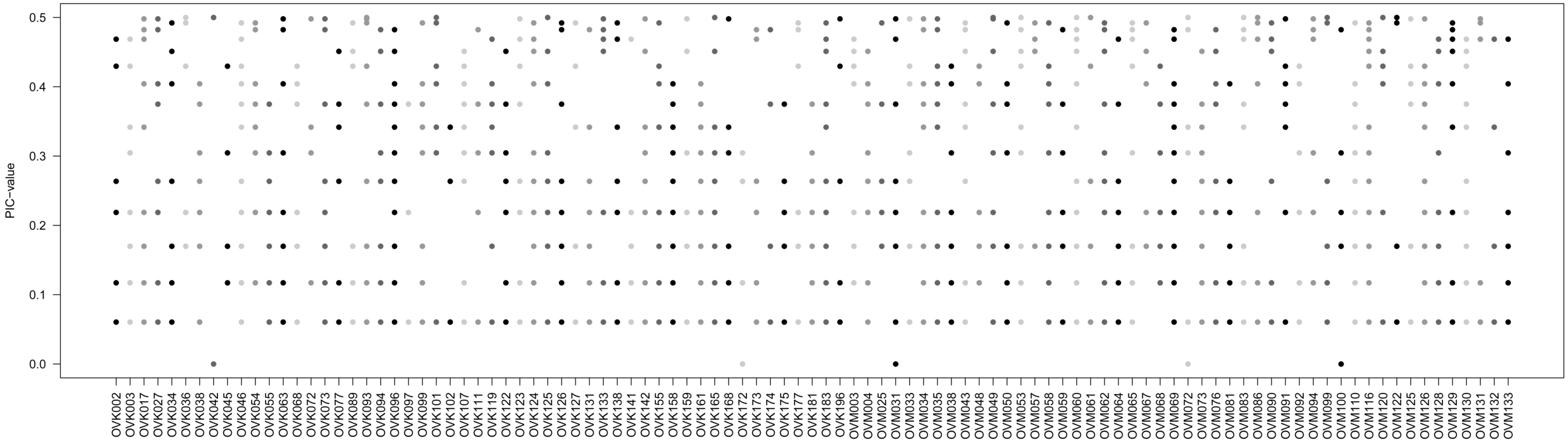

Supplement: Additional file 2: Figure S2. — Polymorphism Information Content (PIC) values for individual alleles at SSR loci. Different grey levels are used for better visual differentiation among alleles of the different SSR markers. (PDF 431 kb) [file 12863_2016_431_MOESM2_ESM.pdf]
